# Entropy and Information Theory in Acoustics

**DOI:** 10.3390/e24121760

**Published:** 2022-12-01

**Authors:** Yuxing Li

**Affiliations:** 1 School of Automation and Information Engineering, Xi’an University of Technology, Xi’an 710048, China; liyuxing@xaut.edu.cn; 2 Shaanxi Key Laboratory of Complex System Control and Intelligent Information Processing, Xi’an University of Technology, Xi’an 710048, China

Acoustics is one of the most studied fields in the 21st century, encompassing underwater acoustics, architectural acoustics, engineering acoustics, physical acoustics, environmental acoustics, psychological acoustics, signal processing in acoustics, and so on. With the application of entropy and information theory in acoustics, many acoustic problems have been solved or improved. Additionally, the current trend seems to indicate that the role of entropy and information theory in the field of acoustics will also steadily increase and lead to many exciting new discoveries. This Special Issue of “Entropy and Information Theory in Acoustics II” aims to collect the latest research results and latest developments in the application of entropy and information theory in the field of acoustics. This collection contains eight papers representing the latest applications of entropy and information theory in the field of acoustics.

In order to extract useful features from nonstationary and nonlinear ship-radiated noise signal (SNS), Xie et al. propose an SNS feature extraction method based on optimized variational mode decomposition, displacement entropy, and normalized Spearman correlation coefficient [1]. The experimental results show that this method can accurately identify five S-RN samples, and the recognition rate is 94%, which is higher than that of other methods.

Li et al. introduce slope entropy (SIEn) into feature extraction in ship-radiated noise signals for the first time, and proposed a single-feature extraction method based on slope entropy and a double feature extraction method based on SIEn combined with permutation entropy [2]. By extracting the features of four measured SNSs, the feasibility of the proposed method is verified. The experimental results show that the proposed SIEn-based single feature extraction algorithm has the highest recognition rate compared with the other four SNS-based single feature extraction methods. The recognition rate of the proposed two-feature extraction method is higher than that of the proposed single-feature extraction method and the other three two-feature extraction methods.

Wasiq et al. propose an intelligent computing paradigm built on a nonlinear autoregressive exogenous (NARX) feedback neural network model with the strength of deep learning, presented for the accurate state estimation of an underwater passive target. To improve tracking accuracy, effective feature estimation, and minimizing the position error of dynamic passive objects, the strength of NARX-based supervised learning is exploited. The root mean square error between the estimated and real position of the passive target in rectangular coordinates is computed for evaluating the worth of the proposed NARX feedback neural network scheme [3].

In order to improve the communication rate of existing bionic concealed-hydroacoustic communication (BC-UAC) and improve the concealment and effectiveness of the constructed bionic communication signal, Xie et al. proposed a bionic covert UAC method based on the time–frequency contour of the bottlenose dolphin whistle, which can overcome the security problems of traditional low-signal–noise-ratio covert communication and exclude detected communication signals such as marine biological noise [4]. The performance of the proposed BC-UAC method, in terms of the Pearson correlation coefficient and bit error rate, is verified under simulated and measured underwater channels.

Based on the theory of fluid–solid coupling, combined with the aeroacoustics computational fluid dynamics method and the frequency domain acoustic wave equation, and using the track–wheel interaction noise software noise simulation prediction model, the rolling aerodynamic noise model of wheel–rail of a high-speed railway is established, and the vibration and noise characteristics of the wheel-rail area of a high-speed railway are analyzed, and the joint simulation and prediction of vibration, rolling noise, and aerodynamic noise of a wheel–rail system are realized. The field test data of the Beijing–Shenyang line is considered to verify the reliability of the model [5]. By analyzing the characteristics of each sound source at different frequencies, corresponding noise reduction measures are proposed.

Wang et al. propose two spectral methods, the Chebyshev–tau and Chebyshev–collocation spectral methods, to solve the normal mode of atmospheric acoustics, and corresponding programs are developed. An artificial absorber layer is added above the atmosphere of interest to reduce the impact of the truncated half-space on the area of interest. Numerical experiments are examined for both downwind and upwind conditions to verify the effectiveness of the methods. The running time data indicated that both spectral methods proposed in this article are faster than the Legendre–Galerkin spectral method proposed previously [6].

Wasiq et al. proposed an application of deep-learning-based neural computing for efficient the real-time state estimation of the Markov chain underwater-maneuvering object [7]. State estimation modeling is developed in the context of bearings-only tracking technology in which the efficiency of the NARX neural network is investigated for ideal and complex ocean environments. Sufficient Monte Carlo simulation results validate the competence of NARX neural computing over conventional generalized pseudo-Bayesian filtering algorithms such as an interacting multiple model extended Kalman filter and an interacting multiple model unscented Kalman filter.

Ma et al. apply the Chebyshev–Galerkin and Chebyshev–collection spectral methods to directly solve the two-dimensional Helmholtz model equation; implement the parallel code of the collection method to effectively improve the calculation effectiveness; and use the analytical solution to verify the feasibility of the two methods for directly solving the two-dimensional ocean acoustic propagation problem [8].

The editors extend their sincerest gratitude toward all of the authors for their excellent contributions to this Special Issue. Special thanks go to all of the reviewers for their time and feedback provided to the authors. Additionally, we sincerely thank the publishers, editors, and all of the members of the *Entropy* editorial board for facilitating this opportunity to present all of these works. 
